# New criteria for breast symmetry evaluation after breast conserving surgery for cancer

**DOI:** 10.1590/0100-6991e-20202698

**Published:** 2021-06-04

**Authors:** RENÉ ALOISIO DA COSTA VIEIRA, GABRIELE BILLER, FABIOLA CRISTINA BRANDINI DA SILVA, JONATHAS JOSÉ DA SILVA, MARCO ANTÔNIO DE OLIVEIRA, ANTÔNIO BAILÃO-JUNIOR

**Affiliations:** 1 - Hospital de Câncer de Barretos, Programa de Pós-graduação em Oncologia - Barretos - SP - Brasil; 2 - Universidade Estadual Paulista (UNESP), Faculdade de Medicina de Botucatu, Programa de Pós-graduação em Tocoginecologia - Botucatu - SP - Brasil; 3 - Hospital de Câncer de Muriaé, Departamento de Cirurgia. Divisão de Mastologia - Muriaé - MG - Brasil; 4 - Hospital de Câncer de Barretos, Núcleo de Apoio ao Pesquisador - Barretos - SP - Brasil; 5 - Hospital de Câncer de Barretos, Departamento de Mastologia e Reconstrução Mamária - Barretos - SP - Brasil

**Keywords:** Breast Neoplasms. Conservative Treatment. Mastectomy, Segmental. Body Image. ROC Analysis, Neoplasia da Mama, Tratamento Conservador, Mastectomia Segmentar, Imagem Corporal, Análise ROC

## Abstract

**Objective:**

to evaluate symmetry after breast-conserving surgery (BCS) for cancer.

**Methods:**

a prospective study of patients undergoing BCS. These patients were photographed using the same criteria of evaluation. The references points used were the nipple height difference (NH), the nipple-manubrium distances (NM), nipple-sternum distances (NS) and the angle between the intramammary fold and the nipple (nipple angle; NA). ImageJ software was used. Three breast symmetry models were evaluated: excellent/others (model 1), excellent-good/others (model 2) and others/poor (model 3). The ROC curve was used to select acceptable criteria for the evaluation of symmetry. Decision tree model analysis was performed*.*

**Results:**

a total of 274 women were evaluated. The BCCT.core result was excellent in 5.8% (16), good in 24.1% (66), fair in 46.4% (127) and poor in 23.7% (65). The difference in NH was associated with good breast area (0.837-0.846); acceptable differences were below 3.1 cm, while unacceptable values were greater than 6.4 cm. Differences in the NM were associated with average breast area (0.709-0.789); a difference in value of less than 4.5 cm was acceptable, while values greater than 6.3 cm were unacceptable. In the decision tree combined model, a good-excellent outcome for patients with differential (d) dNH = 1 (0 to 5.30 cm) and dNM ≠ 3 (<6.28 cm); and for a poor/poor result, values dNM = 3 (> 6.35)*.*

**Conclusions:**

the results presented here are simple tools that can assist the surgeon for breast symmetry evaluation.

## RESUMO

**Objetivo:**

avaliar simetria após a cirurgia conservadora da mama (CCM) para câncer.

**Métodos:**

estudo prospectivo de pacientes submetidos à CCM, as quais foram fotografadas segundo os mesmos critérios de avaliação. Os pontos de referência utilizados foram a diferença de altura do mamilo (AM), a distância mamilo-manúbrio (MM), a distância mamilo-esterno (ME) e o ângulo entre o sulco intramamário e o mamilo (ângulo mamilo; AnM). Foi usado o programa ImageJ. Avaliamos três modelos de simetria mamária: excelente/outros (modelo 1), excelente-bom/outros (modelo 2) e outros/ruim (modelo 3). Aplicamos a curva ROC para selecionar os critérios aceitáveis para a avaliação da simetria. Realizamos análise com o modelo de árvore de decisão.

**Resultados:**

foram avaliadas 274 mulheres. Os resultados do BCCT.core foram excelentes em 5,8% (16), bons em 24,1% (66), regulares em 46,4% (127) e ruins em 23,7% (65). A diferença de AM (dAM) foi associada a boa área mamária (0,837-0,846); diferenças aceitáveis foram inferiores a 3,1 cm, enquanto os valores inaceitáveis foram superiores a 6,4 cm. As diferenças MM (dMM) foram associadas à área regular das mamas (0,709-0,789); diferença de valor inferior a 4,5 cm foi aceitável, enquanto valores superiores a 6,3 cm foram inaceitáveis. O modelo combinado de árvore de decisão demonstrou resultado bom-excelente para pacientes com diferencial (d) dAM = 1 (0 a 5,30 cm) e dMM ≠ 3 (< 6,28 cm), e resultado ruim/ruim com dMM = 3 (> 6,35 cm).

**Conclusões:**

os resultados aqui apresentados são ferramentas simples que podem auxiliar o cirurgião na avaliação da simetria mamária.

## INTRODUCTION

Breast cancer is associated with half of all cancer cases and 38% of cancer-related deaths in developed countries^1^. It is estimated that over 1.7 million new breast cancer cases are diagnosed annually worldwide. Breast-conserving surgery (BCS)^2^ ^,^ ^3^ combined with radiotherapy^2^ ^-^ ^4^ is considered safe. Overall, 57% of women diagnosed in the early stages and 13% of those diagnosed in the late stages of the disease undergo breast-conserving treatment, and most undergo radiotherapy^5^.

For patients who undergo BCS, cosmesis is considered excellent or good in 76.3% and 47% of cases, respectively^6^. The main factors associated with asymmetry are age, higher body mass index and large tumour size^7^. Many patients undergo further breast surgery due to asymmetry, and after the second procedure, 94.5% and 88.8% of patients are satisfied after 1 and 5 years, respectively. However, a second and a third operation are required in 19.1% and 6.4% of cases, respectively^8^.

Women are generally dissatisfied with their breasts, with 42.7% reporting being displeased^9^, and 30% of women who undergo BCS are not satisfied with the aesthetic results^10^. Although there are some parameters for healthy breasts^9^, in cosmetic surgical skin marking, other particular reference points and distances are considered appropriate^11^.

Cosmetic evaluation is very subjective, and inter-examiner correlation is poor. Inter-examiner variation can be minimized after a consensus is reached among examiners, but this is difficult to achieve in clinical practice^12^. Breast Cancer Conservative Treatment Cosmetic Results (BCCT.core) was created to evaluate BCS results^13^ ^,^ ^14^. This software has led to a 70% correlation between examiners^15^. Although BCCT.core is extremely useful and reproducible, this tool is used only in research, which suggests that simpler and more objective breast symmetry evaluation criteria are needed. Health professionals and patients do not always consider the same results to be satisfactory^16^, which indicates that more studies are needed in this area.

## MATERIALS AND METHODS

This was a prospective study that was approved by the Barretos Cancer Hospital Research Ethics Committee (No. 782/2014). A total of 300 patients with breast cancer were randomly and systematically selected at the Mastology and Breast Reconstruction Department - Barretos Cancer Hospital between 05/2015 and 06/2016. Patients were selected based on inclusion criteria, and they agreed to participate in all the study phases that evaluated quality of life and sequelae related to cancer treatment (breast cosmesis, lymphedema, and shoulder mobility) and were interviewed and underwent a rigorous clinical evaluation^17^. This study was supported by “Fundação de Amparo a Pesquisa do Estado de São Paulo (FAPESP; No. 08197-0-0/2014)”, and multiple evaluations were performed. Part of this study (evaluating quality of life^17^ and breast cosmesis^18^) was previously published.

The following patients were considered for inclusion: patients who received treatment exclusively at HCB, those who underwent BCS and radiotherapy for a period exceeding 1 year and patients who provided the written informed consent. Patients with metastatic disease, with recurrence, receiving chemotherapy, with bilateral breast cancer, who were male and with a high number of comorbidities were excluded.

After providing informed consent, the selected patients were taken to a special room containing a background symmetrograph, where points were marked on the sternal manubrium and 20 cm inferiorly. The women were photographed from a distance of 1 metre using a Cyber-Shot DSC-H300 camera with an 8-megapixel resolution. Photographs were obtained bilaterally in an anteroposterior, lateral direction, until the mid-axillary line could be seen, as this is associated with the evaluation of the areolar angle and the pencil drop angle (PDA)^9^. While analysing the photographs, patients whose images were not suitable for evaluation using BCCT.core were excluded, along with patients who underwent central BCS without areolar reconstruction.

**Figure 1:**
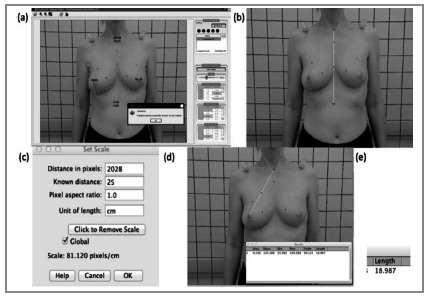
Image parameters. (a) BCCT.core; (b and c) ImageJ calibration; (d, e) analysis and results in cm.

BCCT.core was used for the cosmetic evaluation^14^ ^,^ ^19^. The BCCT.core program performs automatic calculations of different ratios/asymmetries, including the breast volume, skin colour and scarring. The results are given with a 4-point scale (1-excellent, 2-good, 3-fair, and 4-poor)^15^ ^,^ ^19^. These parameters were used as the standard (Figure 1).

**Figure 2:**
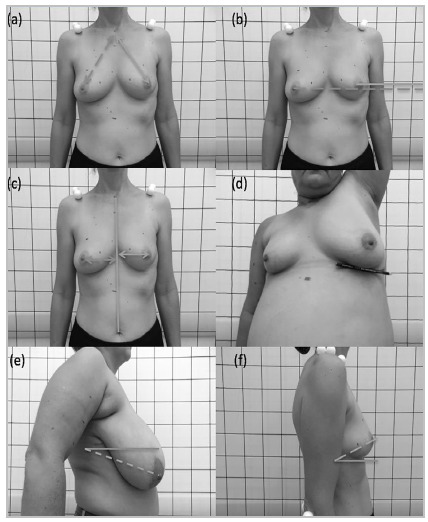
Breast measurement parameters. (a) NM; (b) difference in the NH; (c) NS difference; (d) PDA with arm abduction; (e) negative NA; and (f) positive NA.

The same images were also evaluated with ImageJ software, which was used to evaluate the following distances after calibrating the equipment with known distances: the nipple-manubrium (NM) distance, the nipple angle (NA), the nipple-sternum (NS) distance and the angle of the abducting arm associated with the pencil test (PDA). These measurements were based on a previous study^9^, and for this, the differences of the distances between the nipple-manubrium (NM), the nipple-sternum (NS), the angles between nipples (NA) and the nipple height (NH) were considered. To evaluate the primary breast shape, the contralateral breast (healthy) was evaluated, and ptosis was evaluated with the pencil test (the angle at which a pencil placed under the breast falls with upper limb abduction), the contralateral NM (CNM) and the contralateral NA (CNA). The measurements are shown in figure 2. As there are no criteria in the literature regarding this evaluation, this was considered a pilot study, and we did not perform sample size calculations.

**Figure 3:**
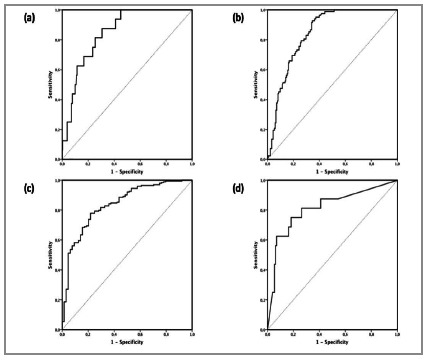
ROC curve for the results: (a) dNM in model 1; (b) dNM in model 2; (c) dNM in model 3; (d) breast shape according to the PDA evaluation with excellent results.

BCCT.core output and ImageJ calculations were first transferred to the IBM SPSS for Mac® program, which was used to perform differential calculations. Subsequently, these data were exported to the MedCalc® program, where the findings related to breast symmetry were dichotomized into excellent/others (model 1), excellent-good/others (model 2) or excellent-good-fair/poor (model 3). The receiver operating characteristic (ROC) curve was used to evaluate the sensitivity, specificity, area and difference in the cut-off point between acceptable and non-acceptable symmetry to identify simple criteria related to good breast symmetry.

From the identified criteria, we opted to analyse those that presented better results for all cut-off points and presented an increasing linearity in relation to the results in the ROC curve. Accordingly, only the difference between the NH and the difference in the NM distance were selected for the construction of a mathematical model. In the IBM SPPS program, a decision tree was used (decision trees; machine learning). The results of BCCT.core were selected and compared with the differences presented above. The CHi-squared Automatic Interaction Detection (CHAID) method was used in two situations: automatic and forcing the inclusion of the two variables in the model. Due to the limited number of patients with excellent criteria, we chose to group the BCCT.core results into excellent-good. In the tree model, the orientation was descending, presenting the classification results according to the association or lack of association of the values.

## RESULTS

Of the 300 patients selected for the study, 3 (1%) patient photographs had inadequate resolution, and 23 (7.7%) underwent central BCS without areolar reconstruction, which resulted in 274 patients who were eligible for inclusion in the study.

The age of the patients ranged from 25.8 to 87.5 years (mean 58.4, standard deviation (SD) 9.8), and patients had undergone breast surgery 1 to 20.2 years prior to the study (mean 6.9; SD 4.1). Overall, 50.4% of tumours were located in the right breast. Tumour sizes ranged from 0.3 to 11 cm (mean 2.4, SD 1.5). Approximately 33.2% of tumours occupied 2 quadrants. The tumours were determined to have the following T-TNM stages: 4.0% Tis, 42.3% T1, 43.1% T2, 7.7% T3 and 2.9% T4. In terms of histology, 87.6% of women had invasive ductal carcinoma, 4.7% had invasive lobular carcinoma, 4.0% had ductal carcinoma in situ, and 3.6% had other breast cancer histologies. All patients underwent BCS and subsequent chest wall radiotherapy. A total of 14.6% (40) of patients underwent procedures with different oncoplastic surgical techniques, and 12% underwent symmetrisation surgeries, of which 9.1%^25^ were concomitant and 2.9%^8^ were performed at a later date.

When the differential values were compared between the sides (Table 1), the sternal-manubrium difference ranged from 0 to 18.8 cm (mean 4.50, SD 3.32), the NH difference ranged from 0 to 21.7 cm (mean 5.40, SD 3.70), the NA difference ranged from 0 to 55.2° (mean 17.1°; SD 12.4°), and the difference between the sternum and nipple ranged from 0 to 10.8 cm (median 2.70, SD 2.10). When the conformational aspects of the contralateral breast were evaluated, it was observed that the PDA ranged from 0 to 180° (mean 142°, SD 47.7°), that the NM distance ranged from 19.2 to 60.0 cm (mean 31.1, SD 6.39) and that the nipple angle ranged from -23.0° to 34.3° (mean 8.22, SD 10.7°). For these parameters, there were data missing for 0 to 11 (4%) patients, with a median of 4 (1.4%) patients.

**Table 1:** Acceptable criteria for the evaluation of breast symmetry after breast-conserving treatment.

	Model	Sensitivity	Specificity	Cut-off	Area under the ROC curve	CI	p
Difference							
	1	87.5	69.3	≤ 3.1 cm	0.846	0.774-0.919	<0.0001
Nipple height (NH)	2	92.7	65.4	≤ 5.3 cm	0.844	0.798-0.889	<0.0001
	3	77.8	78.1	≤ 6.4 cm	0.837	0.781-0.894	<0.0001
	1	93.7	42.5	≤ 4.7 cm	0.682	0.580-0.784	0.014
Nipple-manubrium (NM)	2	89.0	47.9	≤ 5.0 cm	0.709	0.648-0.771	<0.0001
	3	83.9	64.6	≤ 6.3 cm	0.789	0.720-0.857	<0.0001
	1	93.7	40.9	≤ 20.2 cm	0.625	0.525-0.724	0.016
Nipple angle (NA)	2	77.8	46.2	≤ 20.2 cm	0.595	0.524-0.666	0.009
	3	66.8	51.6	≤ 20.6 cm	0.583	0.500-0.666	0.050
	1	-	-	-	0.565	0.414-0.716	0.41
Nipple-sternum (NS)	2	-	-	-	0.562	0.486-0.639	0.110
	3	60.1	68.7	≤ 2.2 cm	0.673	0.600-0.746	<0.0001
Contralateral breast shape							
Pencil drop angle	1	75.0	81.6	≤ 112^o^	0.813	0.689-0.937	<0.0001
	2	72.0	50.7	≤158^o^	0.654	0.582-0.726	<0.0001
	3	53.6	75.4	≤144^o^	0.634	0.562-0.706	0.001
Nipple angle*	1	100.0	48.4	> 6.9^o^	0.736	0.649-0.824	0.002
	2	72.0	57.4	> 8.1^o^	0.676	0.607-0745	<0.0001
	3	51.9	70.3	> 9.1^o^	0.615	0.539-0.692	0.005
Nipple-manubrium	1	75.0	69.4	≤ 27.8 cm	0.733	0.622-0.845	0.002
	2	82.9	44.4	≤ 32.7 cm	0.652	0.582-0.721	<0.0001
	3	68.9	61.5	≤ 32.1 cm	0.684	0.613-0.756	<0.0001

*Model 1 - excellent x others; Model 2 - excellent/good x others; Model 3 - excellent/good/fair x poor; CI - confidence interval.*

*A higher result indicates a more positive value for each test.

According to BCCT.core, the result was excellent in 5.8%^16^ , good in 24.1%^66^, fair in 46.4% (127) and poor in 23.7% (65) of patients.

When the criteria related to differences between measurements were evaluated, 2 analyses attracted our attention: the difference in the NM distance and the difference in the NH distance. According to the NH difference criterion, the results were good regardless of the model used. Measurements below 3.1 cm were considered optimal, and results worsened as the distance increased; the worst results were observed when the difference in this distance was greater than 6.4 cm. In terms of the difference in the NM distance, the results were average, in relation to models 2 and 3, and better results were observed when the difference in this distance was less than 5.0 cm. Worse results were also associated with an increase in this distance, and poorer results were observed with a differential distance greater than 6.3 cm (Table 1).

When the conformational breast data were evaluated and when the contralateral breast served as a reference, a good parameter in relation to the PDA and CNA was observed only in model 1 (excellent x others), while an average relationship was observed with respect to the CNM distance and CNA (Table 1).

In the combined decision tree model (Tables 2 and 3), the estimated decision rule suggested that a good-excellent outcome for patients with differential (d) dNH = 1 (0 to 5.30 cm) and dNM ≠ 3 (<6.28 cm) with a 56.1% probability. For a reasonable result, it suggested that dNH = 2 or 3 (> 5.36 cm) and dNM ≠ 3 (<6.28 cm), with a probability of 68.0%. For a poor/poor result, dNM = 3 (> 6.35), with a 56.0% probability. In the simple model, the dNM variable was excluded from the model. Patients with dNH = 1 (<5.30 cm) presented a 51.6% probability for an excellent/good result, and patients with dNH = 2 or 3 (> 5.36 cm) presented a probability of a poor/poor outcome of 46%.

**Table 2:** Reference values for dNH and dNM.

Model	Points		dNH	dNM
4 points	1	Excellent	3.1 to 0	4.68 to 0
	2	Good	5.30 to 3.32	4.99 to 4.71
	3	Fair	6.40 to 5.36	6.28 to 5.02
	4	Poor	21.66 to 6.46	18.79 to 6.35
3 points	1	Excellent/Good	5.30 to 0	4.99 to 0
	2	Fair	6.40 to 5.36	6.28 to 5.02
	3	Poor	21.66 to 6.46	18.79 to 6.35

d - differential; NH - nipple height; NM - nipple-manubrium.

**Table 3:** Results of the decision tree based on the probability of different combinations.

Model		Prediction			
	Observed	Excellent/good	Fair	Poor	% correct
Simple	Excellent/good	81	0	1	98.8%
	Fair	78	0	45	0
	Poor	16	0	49	75.4%
	% General	64.8%	0%	35.2%	48.1%
Forced	Excellent/good	73	4	5	89.0%
	Fair	51	44	28	35.8%
	Poor	6	17	42	64.6%
	% General	48.1%	24.1%	27.8%	58.9%

## DISCUSSION

Currently, no universal criteria for breast symmetry have been established. The evaluation of the literature related to plastic and reconstructive surgery has revealed some parameters that may be considered appropriate. The reference points are the midclavicular-areola line (20 to 21 cm), the sternal notch-areola line (19 to 24 cm), the areola-sulcus line (4 to 6 cm) and the areola-sternum line (8 to 12 cm)^11^. When normal breasts are evaluated, one should consider patient preferences with respect to their desire for symmetry, and the healthy breast should be evaluated only after the diseased breast has received treatment in an attempt to perform similar procedures.

BCS has acceptable recurrence rates when combined with radiotherapy^2^ ^,^ ^3^ but can be associated with local changes and sequelae^20^. Asymmetry can occur due to the simple absence of symmetrisation at the time of primary surgery and/or as a result of local changes, and the results deteriorate over time^21^.

Oncoplastic surgery for BCS may be used to treat large tumours and can result in wide margins without concomitant increases in complication rates^22^ ^-^ ^24^. It is notable that in this study, oncoplasty was performed in only 14.6% of patients, while symmetrisation was performed in only 12%. This finding suggests that the need for contralateral breast treatment to obtain symmetry should be considered. Of the 23 patients with central tumours, 16 (70%) underwent oncoplastic surgery with a plug-flap, and in the absence of the areola, the BCCT.core calculation may have been impaired, which would have resulted in a reduced incidence of oncoplasty in this study.

A second detail to consider is the long period between the initial surgical procedure and evaluation, which was 6.9 years on average. The breast shape changes over time, and weight increases accentuate differences, especially in the treated breast, because the volume increase in an irradiated breast is smaller than that in an untreated breast due to tissue fibrosis after radiotherapy.

When factors related to breast asymmetry in patients undergoing BCS are evaluated, younger age, bulky tumours^7^ ^,^ ^25^, menopausal status, tumour size, percentage of skin resected, scar orientation^25^, maximum dose of breast radiotherapy^26^, body mass and tumours located in the superomedial and inferolateral quadrant were all associated with greater asymmetry^7^. Patients with marked asymmetry are more likely to want to undergo a breast symmetrisation procedure^7^.

Patient self-evaluations tend to be better than objective findings from a cosmetic point of view^27^. When patient evaluations were compared with objective metric measurements and evaluations by a panel of observers, the key findings were discordant^28^. Current methodologies have attempted to evaluate breast symmetry using three-dimensional calculations^29^ ^,^ ^30^. Soror et al. sought to present a methodology based on the creation of triangles and comparative data between breasts^31^. Studies based on magnetic resonance imaging (MRI)^32^ and three-dimensional technology evaluations^30^ ^,^ ^33^ ^,^ ^34^ are difficult to incorporate in clinical practice. The Breast Analysing Tool (BAT)^21^ software and BCCT.core^35^ were created. Although BCCT.core use has progressively increased^36^, universal criteria for the evaluation of symmetry are lacking. We described simple parameters that can be used. A ROC curve evaluation demonstrated that the results were acceptable when the area was greater than 0.7; these results may be excellent (area≥0.9), good (09>area≥0.8) or average (0.8>area≥0.7). In terms of the differential values between sides, the NM and NH differences should be mentioned. In addition, an increase in distance was associated with poorer results, and both good and average area values were observed. Differential values of 3.1 cm in the NH and 4.7 cm in the NM were associated with excellent results, and these are parameters are easy to use in clinical practice. The remaining methodological differences were not satisfactory.

Regarding the breast shape, PDA values higher than 112°, an NM distance greater than 27.8 cm and an NA of less than 6.9° were associated with more ptosis of the breast, which is associated with worse outcomes. Matthes et al.^9^ sought to establish simple and easy evaluation parameters that were considered acceptable in normal women. They noted that an NM distance of less than 25 cm, a positive nipple to intramammary fold distance and a PDA less than or equal to 90° were associated with 93% patient satisfaction.

The limitations of this study include the lack of patient evaluation before treatment, the long monitoring period, the limited number of patients undergoing symmetrisation and the lack of validation calculations in another patient sample. As this was a pilot study, sample size calculations were not performed, but a convenience sample was used in an effort to identify differential values in a larger sample of patients. Additionally, potential differential values in breast asymmetry were evaluated as opposed to factors related to symmetry. In this regard, patients with excellent results tended to be younger (51.4 years of age, SD 8.5), and the results worsened with increasing age (60.5 years of age, SD 8.8 years with poor outcomes). Furthermore, a proportionately greater number of patients in this group underwent breast symmetrisation (18.8%, p=0.08).

We presented a simple method based on patient photography, which may be used to evaluate BCS results. This method is based on a combination of differences related to the NH and NM distances. Although more studies using the same methodology are necessary, we hope that this simple method will help surgeons in clinical practice.

## CONCLUSION

Using simple points, it was possible to identify parameters that are related to acceptable and unsatisfactory results, which can facilitate the identification and selection of patients for secondary breast correction and symmetrisation surgery.
